# A Rare Presentation of Hepatocellular Carcinoma Infiltrating the Gallbladder

**DOI:** 10.7759/cureus.5140

**Published:** 2019-07-15

**Authors:** Michael Oriakhi, Suhaib A Andrabi, Alabi Olutoyin, Meena Ahluwalia

**Affiliations:** 1 Internal Medicine, Harlem Hospital Center, New York, USA; 2 Hematology / Oncology, Harlem Hospital Center, New York, USA

**Keywords:** liver cancer, metastasis, gall bladder cancer, hepatocellular carcinoma, abdominal and pelvic ct

## Abstract

Hepatocellular carcinoma (HCC) is a solid tumor of the liver and one of the most common primary tumors of the liver. Lifestyle being a major risk factor for the development of HCC makes it a major public health concern worldwide. HCC rarely infiltrates the gall bladder because it rarely destroys the muscle layer and collagen fibers of the gallbladder wall. We report here a rare case of hepatocellular carcinoma that invaded the gallbladder and was initially misdiagnosed as primary gallbladder malignancy invading the liver.

## Introduction

Hepatocellular carcinoma (HCC) is a solid tumor of the liver and one of the most common primary tumors of the liver. Lifestyle being a major risk factor for the development of HCC makes it a major public health concern worldwide [1]. It is one of the leading causes of cancer death and has a high propensity for vascular invasion and metastasis - the lungs, abdominal lymph nodes, and bones being the commonest sites of extrahepatic invasion [2-3].

It is a well-known fact that the HCC sometimes invades the biliary system and since there is no peritoneum between the gallbladder and liver fossa, a gallbladder malignancy can easily invade the liver.

However, HCC rarely infiltrates the gallbladder because it rarely destroys the muscle layer and collagen fibers of the gallbladder wall [4]. We report here a rare case of hepatocellular carcinoma that invaded the gallbladder and was initially misdiagnosed as primary gallbladder malignancy invading the liver.

## Case presentation

The patient is a 70-year-old man, active smoker, with a medical history of human immunodeficiency virus (HIV) on highly active antiretroviral therapy (HAART), hypertension and asthma who presented to our emergency room with complaints of worsening abdominal pain of several months and an episode of melena.

On examination he was alert and oriented and in mild distress; cardiovascular, respiratory and neurologic examination was unremarkable with tenderness and guarding in the right upper quadrant.

Complete blood count revealed hemoglobin of 17 g/dL and hematocrit of 53%; liver function test showed aspartate aminotransferase (AST) of 215 U/L, alanine aminotransferase (ALT) of 64 U/L, alkaline phosphatase (ALP) 252 U/L, total protein 8.6 g/dL, total bilirubin 3.3 mg/dL, direct bilirubin 2.6 mg/dL, albumin 3.9 g/dL; Alpha-fetoprotein (AFP) 150981.3 ng/mL, hepatitis C virus antibody positive, carcinoembryonic antigen (CEA) <0.5 ng/mL, and cancer antigen (CA 19-9) 120.5 U/mL.

Abdominal ultrasound revealed hepatomegaly, multiple echogenic metastases within the liver, either of hepatic origin or metastases from the gallbladder. Intrahepatic biliary radicle dilatation is present. Abnormal gallbladder compatible with primary gallbladder carcinoma versus metastases from the liver.

**Figure 1:**
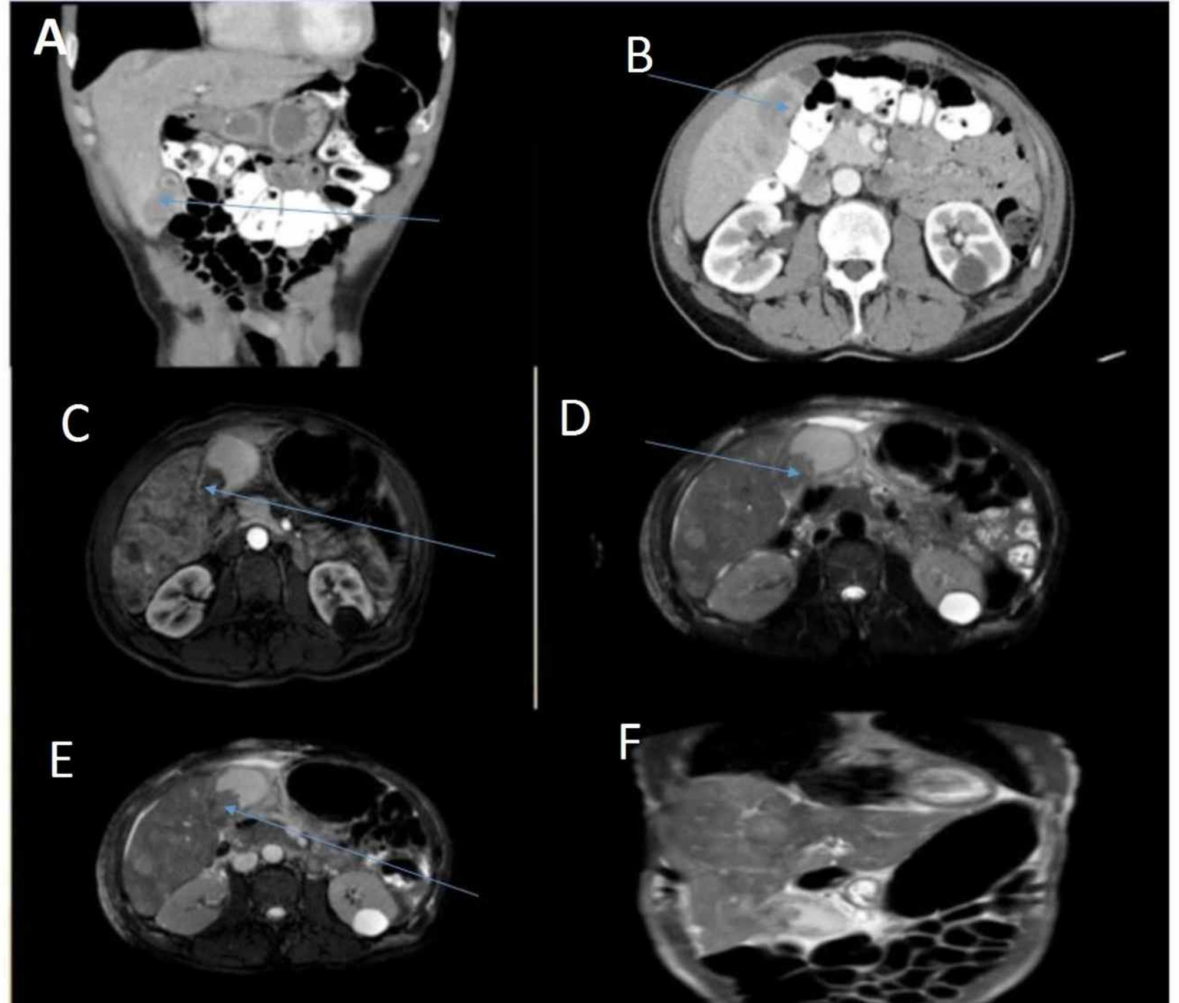
Abdominal computed tomography (A and B) and magnetic resonance cholangiopancreatography (C to F) Innumerable complex, thick walled, heavily separated ill-defined T2 hyperintense avidly enhancing masses scattered throughout the liver suspicious for metastatic cancer (blue arrows - A and B). A small volume ascites to correlate with histopathology. A 3.5 x 2.5 x 2.9 cm mass within the gallbladder lumen (blue arrows - C to F).

Abdominal computed tomography (CT) scan was done with the impression of findings highly suspicious for gallbladder carcinoma with diffuse neoplastic invasion of the liver as described above. Magnetic resonance cholangiopancreatography (MRCP) done revealed the following impression: 3.5 x 2.5 x 2.9 cm mass within gallbladder lumen along with innumerable complex, thick walled, heavily septated ill-defined T2 hyperintense avidly enhancing masses scattered throughout the liver suspicious for metastatic cancer. Small volume of ascites to correlate with tissue histopathology (Figure 1).

**Figure 2:**
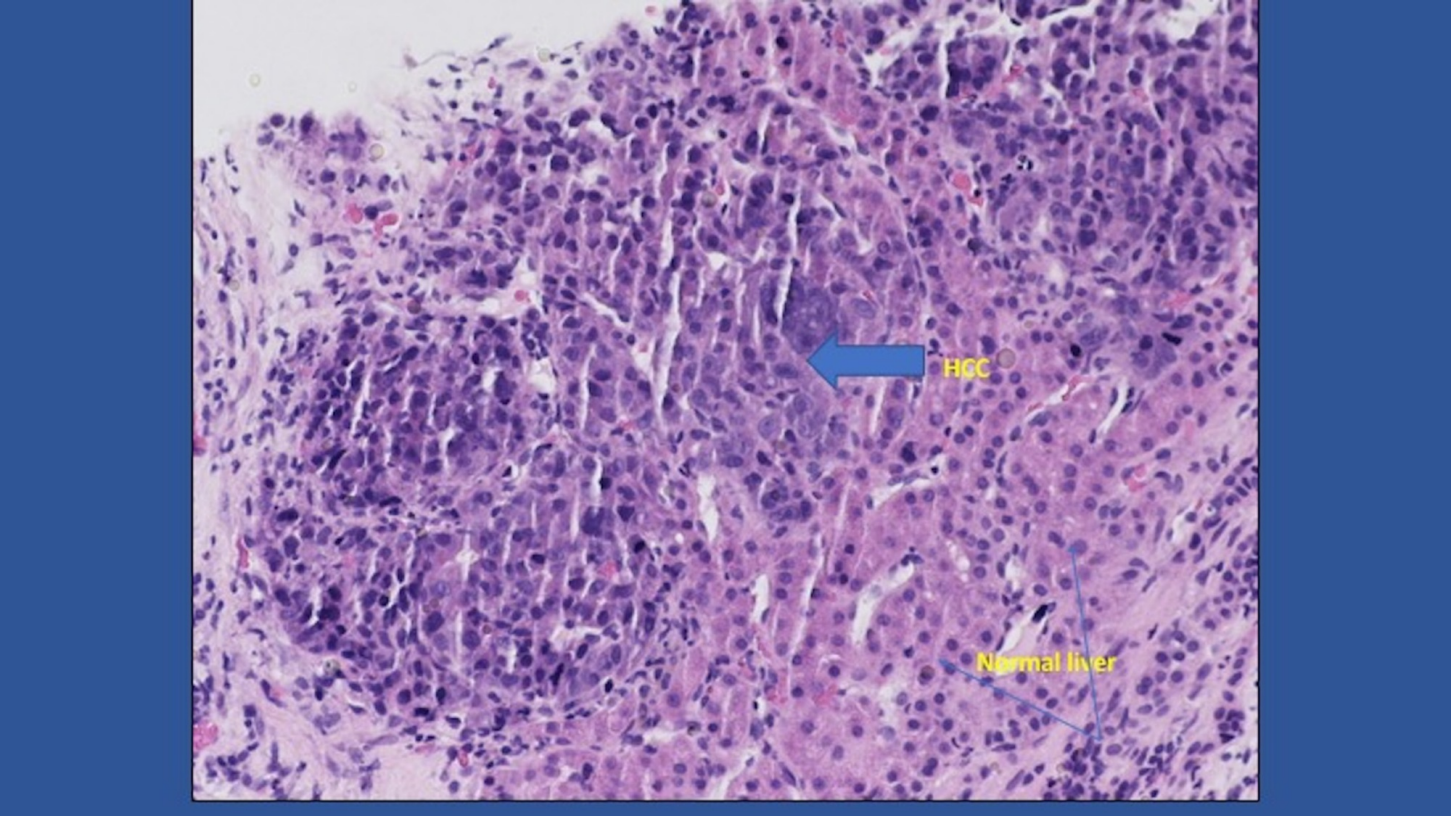
Hepatocellular carcinoma (HCC) foci (solid arrow) and normal liver (line arrow) (H&E x 40)

**Figure 3:**
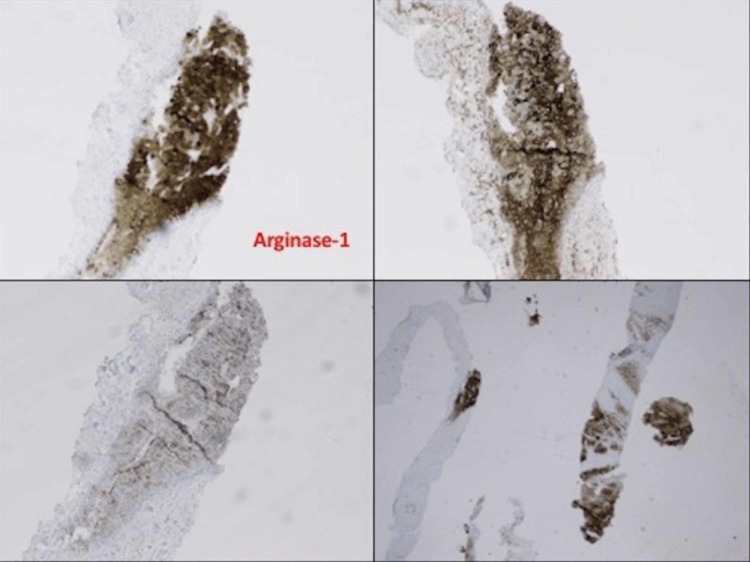
Liver biopsy histopathology showing liver tissue replacement by tumor/hepatocellular carcinoma

Interventional radiology-guided biopsy was done with multiple core biopsy specimen taken and delivered to pathology for analysis. Pathology result was hepatocellular carcinoma (Figures 2, 3).

## Discussion

HCC is a solid tumor of the liver and one of the most common primary tumors of the liver. Lifestyle being a major risk factor for the development of HCC makes it a major public health concern worldwide [1]. Most of the patients who end up developing HCC, would have had a background of established liver cirrhosis with varying risk factors. Viral hepatitis (hepatitis B and C), chronic alcoholism, and nonalcoholic fatty liver disease are the most common risk factors worldwide [1,5]. It is one of the leading causes of cancer death and has a high propensity for vascular invasion and metastasis at the time of diagnosis. It has the propensity for both intrahepatic and extrahepatic invasion. The lungs, abdominal lymph nodes, and bones are the commonest sites of extrahepatic invasion [2-3]. The incidence of metastasis to the gallbladder is extremely rare and there are only case reports or case series available in literature [2,6]. HCC commonly shows intrahepatic spread as compared to extrahepatic malignancy. However, there have been a few clinical reports of cases with metastatic tumor of the gallbladder reported [6]. It is a well-known fact that the HCC sometimes invades the biliary system and since there is no peritoneum between the gallbladder and liver fossa, a gallbladder malignancy can easily invade the liver. However, HCC rarely infiltrates the gallbladder because it rarely destroys the muscle layer and collagen fibers of the gallbladder wall [4]. Imaging techniques play a very important role in the detection, characterization, staging, surveillance, and prognosis of HCC. Screening strategy for patients with chronic liver disease cirrhosis includes the determination of serum α-fetoprotein (AFP) levels and an abdominal ultrasound every six months to detect HCC at an earlier stage [7].

Contrast-enhanced magnetic resonance imaging (MRI) and helical CT scan are the best imaging techniques currently available for the noninvasive diagnosis of hepatocellular carcinoma [1,5]. The most effective curative therapy is liver transplantation and the five-year survival rates for patients undergoing curative therapies (liver transplant, hepatic resection, and percutaneous ablative techniques) range between 40 and 75% and this is limited by the stage at which the tumor is detected. Treatment options for metastatic diseases include chemoembolization, systemic chemotherapy, and palliative care [1,5]. The patient was educated on the diagnosis and stage of the disease and he opted for palliative care.

## Conclusions

With this, we hope to remind clinicians, radiologists, and surgeons, that HHC infiltrating the gallbladder, howbeit rare, should be included in the differential diagnosis of patients presenting with a gallbladder mass in imaging studies.
